# In vivo assessment of canine muscle adiposity by real time ultrasonography and image analysis

**DOI:** 10.1186/1751-0147-57-S1-O2

**Published:** 2015-09-25

**Authors:** Rita Payan-Carreira, Luis Martins, Sónia Miranda, Pedro O Pinto, Severiano R Silva

**Affiliations:** 1 Animal and Veterinary Research Center, CECAV, Universidade de Trás-os-Montes e Alto Douro, Vila Real, Portugal; 2 Escola Universitária Vasco da Gama, Coimbra, Portugal; 3 Hospital Veterinário do Baixo Vouga, Águeda, Portugal

## Introduction

Obesity in dogs is a growing concern. Different surveys report a recent increase in dog obesity, their estimates ranging from 22 to over 50%. Surveillance of dog body condition score (BCS) is a routine practice but it is not sensitive enough. Ultrasound (US) assessment of body fat deposits was previously used in different species to predict adiposity, and shows several advantages, like its relative low cost, portability, and great repeatability. In human, intramuscular fat was associated with obesity systemic effects.

## Objectives

This study aimed to assess the relationship between BCS and intramuscular fat infiltration (IMF) in healthy dogs.

## Material and methods

Twenty-eight dogs were used, with different sizes (nain-4; small-10; medium-14), weights (5.2–33.0 kg) and BCS (2-4 in a 5 points scale). US images were collected from non-sedated dogs in right lateral recumbency and were analysed in Image J. The rough surface and small size of IMF flecks within the muscle cause sound waves to scatter, which produced hyperechoic speckles on the US image. An IMF threshold value is determined. Thereafter a region of interest is converted into an 8-bit grey scale and the threshold applied. The IMF-related mask was overlaid onto the image and numerical data extracted. Relationships between IMF (dependent) and BCS (independent) were established by simple and polynomial regressions.

## Results

The coefficient of determination in polynomial equations was always higher than or equal to that of linear equations (r2 between 0.197, p=0.018, and 0.811, p<0.001).

## Conclusion

This study showed that pertinent information about dog IMF depot might be obtained from US image analysis.

